# Improving iSpyMacCas9 multiplex genome editing in rice by CRISPR‐combo‐mediated *BBM1* activation

**DOI:** 10.1111/tpj.70980

**Published:** 2026-06-07

**Authors:** Innocent Byiringiro, Danyel Fernandes Contiliani, Colin Davies, Filiz Gurel, Silvana Creste, Yiping Qi

**Affiliations:** ^1^ Department of Plant Science and Landscape Architecture University of Maryland College Park Maryland USA; ^2^ Graduate Program of Genetics, Ribeirao Preto Medical School University of Sao Paulo Ribeirao Preto SP Brazil; ^3^ Sugarcane Center Agronomic Institute (IAC) Ribeirao Preto SP Brazil; ^4^ Institute of Bioscience and Biotechnology Research University of Maryland Rockville Maryland USA

**Keywords:** technical advance, CRISPR‐combo, Cas12b, iSpyMacCas9, targeted mutagenesis, transcriptional activation, OsBBM1

## Abstract

The recently developed CRISPR‐Combo technology enables simultaneous targeted mutagenesis and transcriptional activation in plants. However, its reliance on SpCas9 limits its use at AT‐rich genomic loci, such as promoter regions commonly targeted for transcription activation. To overcome this limitation, we explored the usage of Cas12b and iSpyMacCas9 in the CRISPR‐Combo architecture for simultaneous genome editing and gene activation. We tested these expanded CRISPR‐Combo systems for hormone‐free regeneration of rice plants by transcriptional activation of a morphogenic gene, *OsBBM1*, while knocking out the genes of interest. The Cas12b‐Combo system induced mild *OsBBM1* upregulation (~3‐fold), which did not affect the genome editing efficiency. By contrast, iSpyMacCas9‐Combo achieved approximately 12‐fold *OsBBM1* transcriptional activation, supporting hormone‐free regeneration at a high rate (42%). As a result, iSpyMacCas9‐Combo conferred higher genome editing efficiency, including improved multiplex editing, than the standard iSpyMacCas9 system, either with or without hormones during rice regeneration. Hence, our data prove iSpyMacCas9‐Combo to be a more efficient system for genome editing in rice, especially at low‐efficiency target sites, when coupled with *OsBBM1* transcriptional activation. These findings establish iSpyMacCas9‐Combo as a useful addition to the CRISPR‐Combo toolkit, expanding its genomic targeting scope and enabling more efficient genome editing by activation of an appropriate endogenous gene such as *OsBBM1* in rice.

## Significance Statement

This study establishes iSpyMacCas9‐Combo as an expanded CRISPR‐Combo platform that enables efficient simultaneous genome editing and transcriptional activation in rice, particularly at AT‐rich or low‐efficiency target sites. By activating endogenous *OsBBM1*, iSpyMacCas9‐Combo supports hormone‐free regeneration and improves multiplex genome editing efficiency, broadening the utility of CRISPR‐Combo for plant genome engineering.

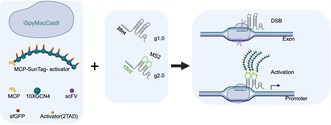

## INTRODUCTION

The CRISPR‐Cas system has been harnessed for genetic engineering such as genome editing (Feng et al., 2013; Jinek et al., 2012) and gene transcriptional regulation (Gilbert et al., 2014; Lowder et al., 2015; Piatek et al., 2015). CRISPR knockout (CRISPRko) can be carried out by RNA‐guided site‐specific nucleases, such as Cas9 (Jinek et al., 2012) and Cas12a/b (Bernabé‐Orts et al., 2019; Ming et al., 2020), which generate DNA double‐strand breaks (DSBs) that are repaired predominantly by the error‐prone non‐homologous end‐joining repair pathway (Cong et al., 2013; Fauser et al., 2014). On the other hand, CRISPR activation (CRISPRa) is typically based on deactivated Cas (dCas) proteins fused to or recruiting activation domains, which induce gene expression (Gilbert et al., 2013; Gilbert et al., 2014; Pan, Sretenovic, & Qi, 2021; Pan, Wu, et al., 2021). Both approaches have been extensively optimized and applied to several plant species (Li et al., 2013; Shan et al., 2013; Fauser et al., 2014; Čermák et al., 2017; Ming et al., 2020; Pan, Wu, et al., 2021; Pan et al., 2022; Byiringiro et al., 2024).

Recently, an orthogonal system was reported – CRISPR‐Combo – combining CRISPRko and CRISPRa in the same approach to enable simultaneous targeted mutagenesis and transcriptional activation in plants (Pan et al., 2022). The key element of CRISPR‐Combo was elaborated using traditional (20 nt) and truncated (15 nt) single‐guide RNA (sgRNA) protospacers, the latter being unable to generate DSBs, even when complexed with Cas9 (Kiani et al., 2015). Additionally, an engineered sgRNA scaffold (2.0) containing two MS2 RNA aptamers for recruiting transcriptional activators was coupled with the truncated sgRNA protospacers. CRISPR‐Combo can be used to promote plant regeneration and augment genome editing efficiency in plants. For instance, the CRISPR‐Combo system demonstrated its usefulness in hormone‐free (HF) regeneration of multiplex gene‐edited rice lines by upregulating *BABY BOOM 1* (*OsBBM1*) gene expression (Pan et al., 2022). The original paper reported CRISPR‐Combo systems based on wild‐type SpCas9 and PAM‐less SpRY (Pan et al., 2022). However, since SpCas9 recognizes 5’‐NGG‐3′ as a protospacer adjacent motif (PAM), it has a limited scope when targeting promoter regions for transcriptional activation, as these regions often display high AT content. Although SpRY can broaden targeting scope, its derived CRISPR‐Combo system was not efficient for genome editing and gene activation (Pan et al., 2022). Thus, adopting AT‐targeting Cas nucleases in CRISPR‐Combo systems is still warranted.

Therefore, this work aimed to expand the scope of CRISPR‐Combo systems by employing Cas12b (formerly known as C2c1) and iSpyMacCas9 nucleases, which recognize VTTV (V = A/C/G) and NAAR (R = A/G) PAM sites, respectively (Chatterjee et al., 2020; Ming et al., 2020; Sretenovic et al., 2021; Strecker et al., 2019). We assembled and tested several constructs for simultaneous transcriptional activation and multiplex gene editing in rice stable lines. We specifically explored the idea of *OsBBM1* transcriptional activation to improve genome editing efficiency in rice. By developing and comparing these new orthogonal systems in rice, we benchmarked iSpyMacCas9‐Combo as a promising CRISPR‐Combo system in plants.

## RESULTS

### Developing and testing a Cas12b‐based CRISPR‐combo system

**Figure 1:**
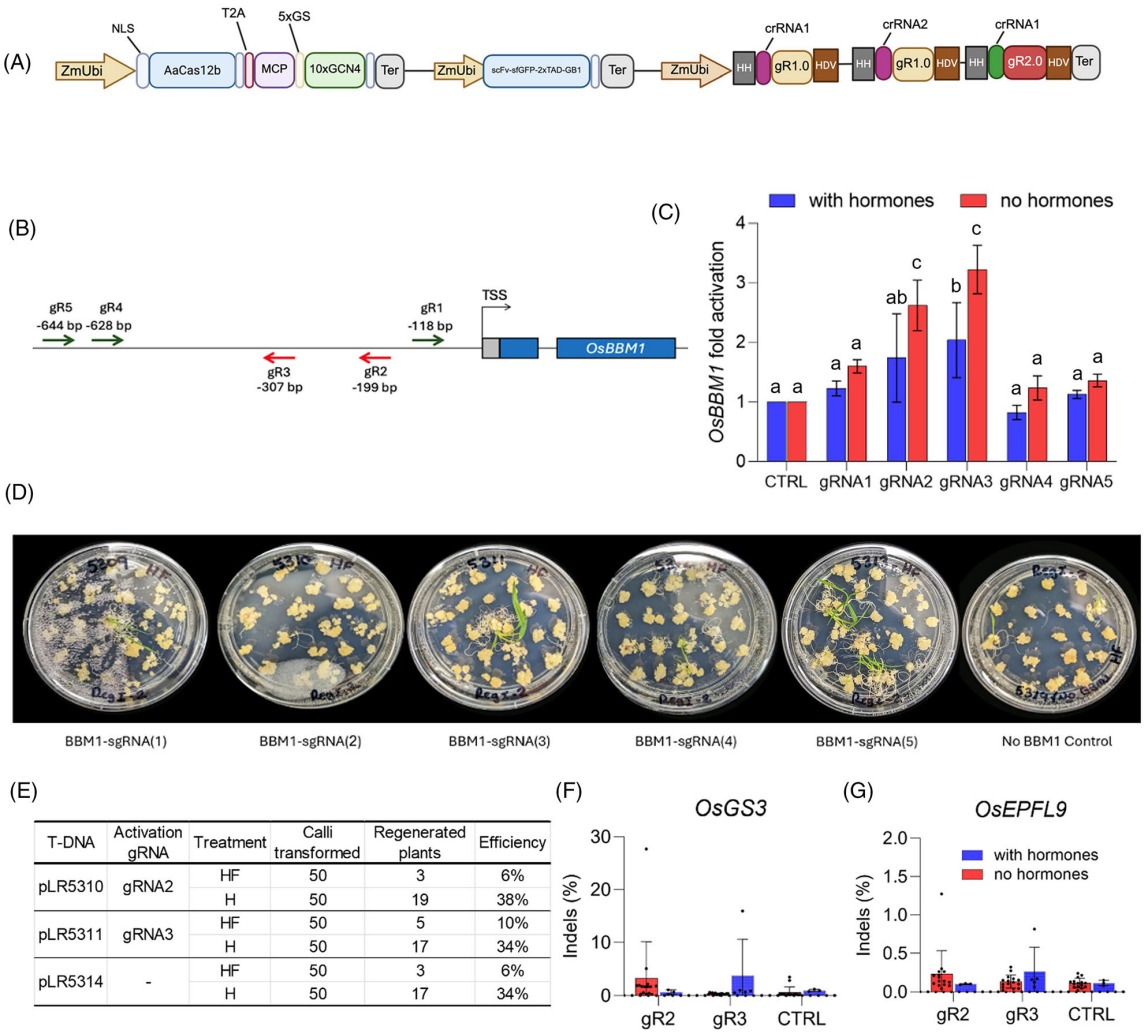
Development of the Cas12b‐Combo system for simultaneous targeted mutagenesis and transcriptional activation. (A) Schematic illustration of the Cas12b‐Combo T‐DNA expression vector. NLS, nuclear localization signal; GS, glycine‐serine linker; SunTag, 10 tandem repeats of GCN4 peptide; Ter, terminator; HH, hammerhead. (B) Designed gRNA2.0 positions around the *OsBBM1* promoter region. TSS, transcription start site. (C) Cas12b‐Combo‐mediated transcriptional activation of *OsBBM1* in rice calli measured by RT‐qPCR. CTRL samples are calli transformed with T‐DNA vectors lacking the *OsBBM1* activating guide. Error bars indicate mean ± SD (*n* = 3 independent experiments). *P*‐values were obtained using a two‐tailed Student's *t*‐test and lowercase letters indicate statistical differences. (D) Representative rice calli following 4 weeks of regeneration under hormone‐free conditions. (E) Number of regenerated plants per construct and transformation efficiencies under hormone‐free (HF) and hormone‐supplemented (H) conditions. (F, G) Indel frequencies at the *OsGS3* (f) and *OsEPFL9* (g) target sites in independent T0 lines. Each dot represents one T0 line.

Although the CRISPR‐Combo system was previously developed based on CRISPR‐Cas9 (Pan et al., 2022), we reasoned that we could develop a CRISPR‐Combo system based on Cas12b to target VTTV PAMs. Among the previously tested Cas12b nucleases, the Cas12b from *Alicyclobacillus acidiphilus*, AaCas12b, displays higher targeted mutagenesis rates in rice stable lines with no detectable off‐target effects (Ming et al., 2020; Gurel et al., 2023). Considering the engineered sgRNA scaffold (2.0) used in the CRISPR‐Combo system, Cas12b would be a better choice for this approach as it also requires a CRISPR RNA (crRNA) and a trans‐activating crRNA (tracrRNA), which make it more amenable than Cas12a, for instance. The construct system contains an AaCas12b expression cassette fused to a SunTag array by a T2A self‐cleaving peptide. The system employs two types of sgRNAs: g1.0, which uses a standard scaffold for genome editing, and g2.0, which incorporates MS2 aptamer loops for recruitment of the SunTag‐transcriptional activator complex (Figure 1A; Figure S1). Two genome editing gRNAs in g1.0 and one activation gRNA in gR2.0 were multiplexed based on the previously established gRNA expression system (Ming et al., 2020). To evaluate the effectiveness of this system for activating gene expression, five g2.0 sgRNAs were designed to target distinct regions of the *OsBBM1* promoter (Figure 1B), cloned into separate T‐DNA vectors and transformed into rice calli through *A. tumefaciens* infection (Figure S3). After 4 weeks of rice calli selection, significant activation of *OsBBM1* gene expression, as assessed by RT‐qPCR, was observed only for gRNA2‐ and gRNA3‐transformed calli (Figure 1C), whereas the other guides (gRNA1, gRNA4, gRNA5) showed little to no activation (Figure 1C). The most effective guide, gRNA3, induced approximately a threefold increase in *OsBBM1* transcript levels compared with no‐guide control, while gRNA2 yielded a modest increase (~2‐fold). The lack of activation by gRNA4 and gRNA5 was not unexpected, as their target sites are located outside the optimal window for CRISPRa (Pan, Wu, et al., 2021). Additionally, calli maintained on HF medium exhibited a higher *OsBBM1* upregulation (Figure 1C,D).

Despite achieving mild *OsBBM1* activation, the Cas12b‐Combo system did not substantially improve regeneration as previously observed with SpCas9‐based CRISPR‐Combo systems (Pan et al., 2022). Fewer plants were regenerated from calli grown on HF medium across all constructs and showed no clear correlation with *OsBBM1* expression levels (Figure 1E), indicating negligible effects in regeneration by modest activation of *OsBBM1*. Consistent with regeneration levels, targeted mutagenesis efficiency in Cas12b‐Combo lines remained low, as revealed by next‐generation sequencing (NGS) of PCR amplicons. Indel frequencies at the two multiplexed target genes, *OsGS3* and *OsEPFL9*, were generally below 10% in T_0_ plants across all constructs (Figure 1F,G). These results indicate that Cas12b‐Combo can achieve modest *OsBBM1* activation at the A/T‐rich target sites. However, such a low level of *OsBBM1* transcriptional activation did not translate to improved plant regeneration and genome editing.

### Developing and testing iSpyMacCas9‐combo for simultaneous genome editing and gene activation

**Figure 2:**
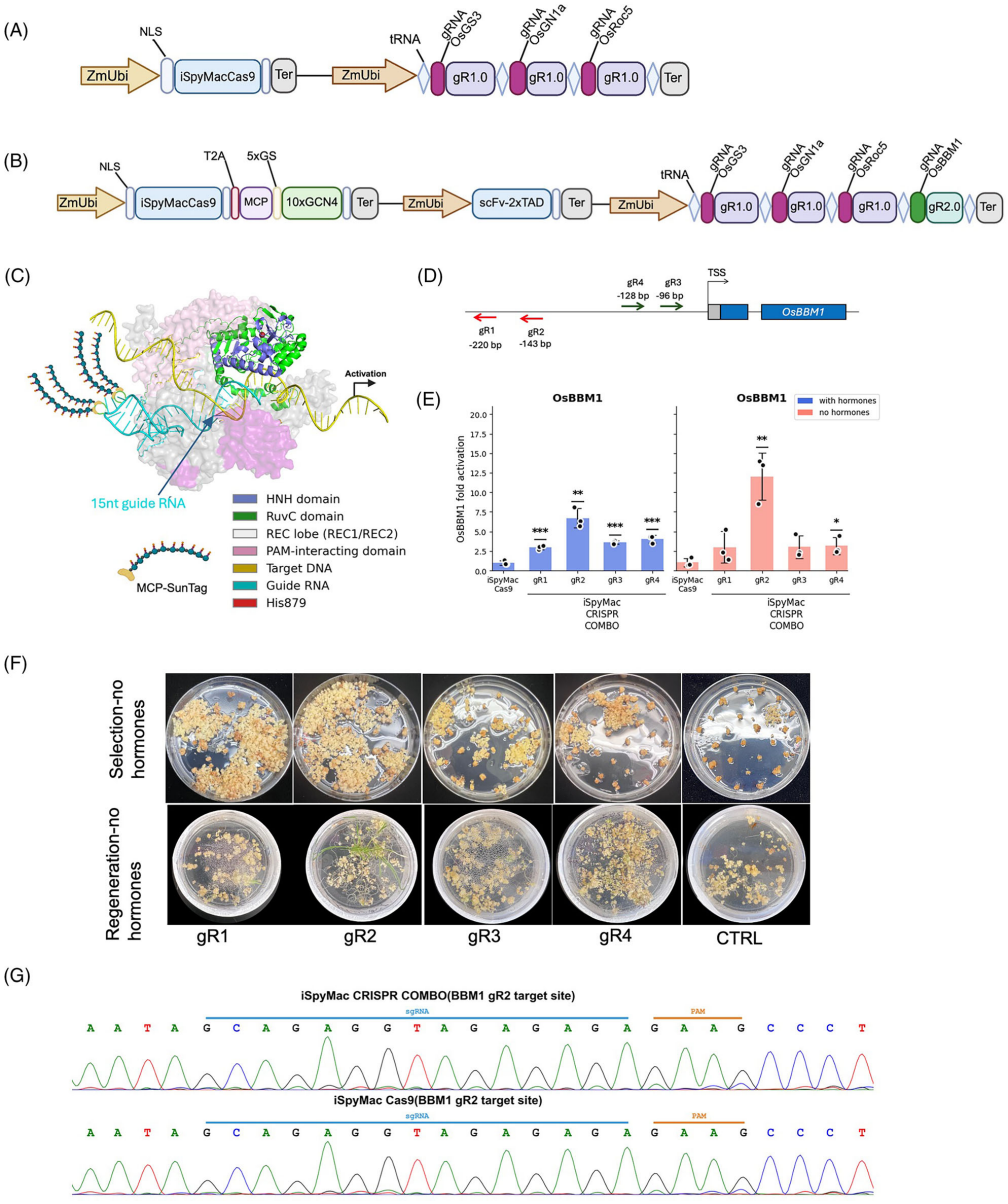
Development of an iSpyMacCas9‐based CRISPR‐Combo system and evaluation of OsBBM1 activation in rice. (A) Schematic of the iSpyMacCas9‐ (control) T‐DNA vector, expressing iSpyMacCas9 with nuclear localization signals (NLS), and transcriptional terminators (Ter). (B) Schematic illustration of the iSpyMacCas9 CRISPR‐Combo T‐DNA expression vector; sgRNAs are expressed as polycistronic RNA and processed by tRNA. (C) AlphaFold3‐based structural model of iSpyMacCas9 bound to a 15‐nt truncated activation guide RNA and target DNA (domain colors: HNH, slate blue; RuvC, green; PAM‐interacting, pink; REC lobe/REC1/REC2, gray; target DNA, yellow; guide RNA, cyan; His879, red). The HNH catalytic residue His879 is located approximately 41–43 Å from the scissile phosphate well outside the ~6–7 Å required for productive cleavage indicating that the truncated guide supports stable Cas9 recruitment and target engagement without positioning the nuclease domains for DSB formation. These poses are illustrative docking models; see Figure S2 for a side‐by‐side comparison with the cleavage‐competent 20‐nt guide conformation. (D) Locations of four candidate *OsBBM1* promoter‐targeting activation guides (gR1‐gR4) relative to the *OsBBM1* transcription start site (TSS). (E) RT‐qPCR quantification of *OsBBM1* transcript levels in rice calli transformed with each activation guide construct (gR1‐gR4) or the corresponding control. Bars represent mean ± SD from *n* = 3 independent biological repeats; two‐tailed Student's *t*‐test (*P* < 0.05; *P* < 0.01; *P* < 0.001). (F) Representative callus and regeneration phenotypes under hormone‐free selection/regeneration conditions for each *OsBBM1* activation guide construct (gR1–gR4) and the control. (G) Representative Sanger sequencing traces spanning the *OsBBM1* activation guide binding region showing no detectable indels, supporting activation without measurable editing at the activation target site.

Given the limited performance of Cas12b‐Combo, we next evaluated iSpyMacCas9 as an alternative CRISPR‐Combo system for A/T‐rich sequences. The iSpyMacCas9 nuclease preferentially recognizes NAAR PAMs and confers efficient genome editing in rice (Sretenovic et al., 2021) and mammalian cells (Chatterjee et al., 2020). We coupled the iSpyMacCas9 nuclease to the CRISPR‐Combo architecture, expressing four gRNAs in the tRNA expression cassette (Figure 2A,B). Here, we targeted *OsGS3*, *OsROC5*, and *OsGN1a* for mutagenesis and *OsBBM1* for transcriptional activation. To enable transcriptional activation without cleavage, *OsBBM1* promoter gRNAs were designed with 15 nt spacers, which recruit the activator complex but are insufficient for nuclease activation and DSB formation (Sternberg et al., 2015; Dagdas et al., 2017; Kiernan *et al*., 2025). To visualize the truncated sgRNA‐DNA iSpyMacCas9 complex, we used AlphaFold3 (Figure 2C; Figure S2). For *OsBBM1* activation, we designed four gRNAs across the promoter region (Figure 2D), focusing on the optimal window for transcriptional activation (Pan, Sretenovic, & Qi, 2021; Pan, Wu, et al., 2021). Each activation gRNA, in the gR2.0 scaffold, was cloned into a separate T‐DNA vector, along with a negative control lacking an *OsBBM1* activation gRNA.

These constructs were tested in rice stable transformation with two calli selection and regeneration conditions: with hormones and no hormones (Pan et al., 2022). After 6 weeks in rice selection medium, actively proliferating calli samples were collected for gene expression assessment via RT‐qPCR. At this stage, significant *OsBBM1* activation was observed in gR2‐transformed calli (~12‐fold versus control) (Figure 2E). Moreover, accelerated regeneration was observed for this iSpyMacCas9‐Combo construct under HF conditions (Figure 2F). Sanger sequencing confirmed that no edits were detected at the targeted *OsBBM1* promoter site in randomly selected lines (Figure 2G), supporting that 15 nt spacers could not lead to genome editing, as expected.

After the tissue culture process, we counted the number of regenerated plants for each construct in different treatments (Table S1). Under HF regeneration, gR2 achieved 42% regeneration efficiency, comparable to its control group (with hormones) with 38% of regeneration. The gR4 also enabled more than 20% of HF regeneration. Under HF conditions, regeneration was entirely dependent on *OsBBM1* activation; transformed calli lacking the activation gRNA typically failed to regenerate, whereas *OsBBM1*‐gR2 lines regenerated efficiently. Collectively, these data suggest that some of the iSpyMacCas9‐Combo constructs conferred higher levels of *OsBBM1* activation that led to improved regeneration. Although gR1, gR3, and gR4 significantly increased *OsBBM1* transcript levels, especially under the hormone‐supplemented condition (Figure 2E), this moderate level of activation did not translate into increased plant regeneration (Table S1). This suggests that although *OsBBM1* activation supports regeneration, its transcript abundance might need to meet and surpass a threshold to have this effect. Consistent with the data from the Cas12b‐Combo constructs, twofold to threefold activation of *OsBBM1* appeared to be still below such a threshold.

### iSpyMacCas9‐combo enhances multiplex editing efficiency by *OsBBM1* activation

**Figure 3:**
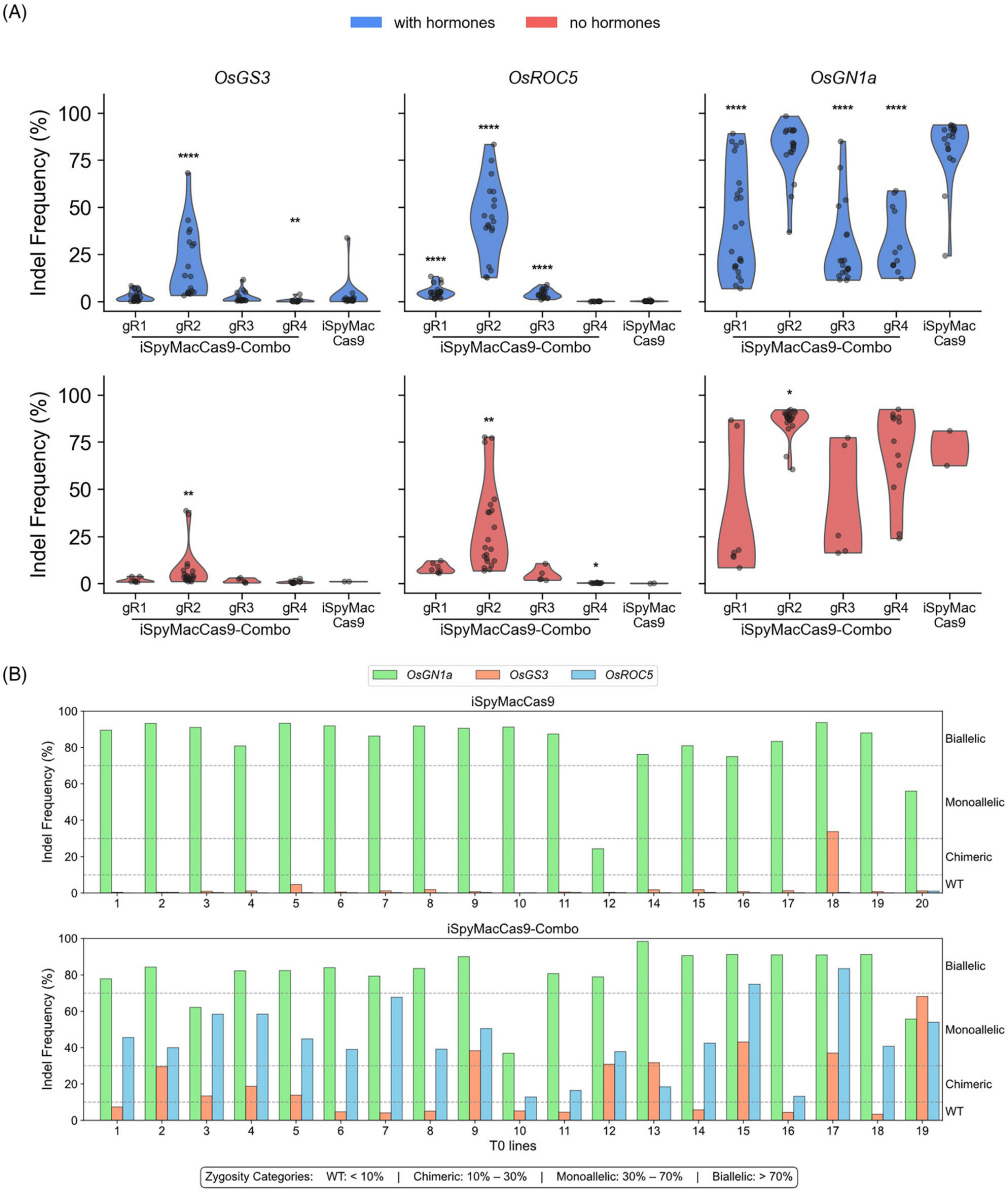
Enhanced multiplexed genome editing efficiency by OsBBM1 activation in rice with iSpyMacCas9 CRISPR‐Combo. (A) Indel frequencies at *OsGS3*, *OsROC5*, and *OsGN1a* in independent T0 lines transformed with iSpyMacCas9‐Combo constructs carrying four *OsBBM1* activation guides (gR1‐gR4) or the standard iSpyMacCas9 control, under hormone‐supplemented (blue, top) or hormone‐free (red, bottom) regeneration conditions. Each dot represents one T0 line. Asterisks denote statistical significance versus iSpyMacCas9 by two‐sided Mann–Whitney U test (*P < 0.05; **P < 0.01; ****P < 0.0001); comparisons not reaching significance are not shown. iSpyMacCas9‐Combo with *OsBBM*1‐gR2 significantly improved editing at *OsGS3* and *OsROC5* under both conditions. Per‐line editing outcomes (indel frequencies) at *OsGN1a*, *OsGS3*, and *OsROC5* in independent T0 lines regenerated with or without *OsBBM1* activation. (B) Summary of multiplex editing outcomes (single/double/triple mutants) comparing lines without *OsBBM1* activation to lines with *OsBBM1* activation (gR2) on hormone‐supplemented medium, summarized by zygosity class. Zygosity thresholds: indel frequency <10% (WT); 10–30% (chimeric); 30–70% (monoallelic); >70% (biallelic).

Based on our previous observation, SpCas9‐Combo enriched genome editing events via *OsBBM1* activation (Pan et al., 2022). To test whether the iSpyMacCas9‐Combo could similarly confer higher genome editing efficiency than the standard iSpyMacCas9 system, we used NGS amplicon sequencing to analyze the editing outcomes at the three target sites among the transgenic plants. We found iSpyMacCas9 barely generated genome editing at *OsGS3* and *OsROC5* sites in both plant regeneration conditions (Figure 3A). However, iSpyMacCas9‐Combo with *OsBBM1* activation by gR2 resulted in significantly higher genome editing efficiency at both sites, whereas other iSpyMacCas9‐Combo constructs (gR1, gR3, and gR4) failed to generate efficient editing (*P* < 0.05; Figure 3A). At *OsGN1a* site, all constructs generated detectable editing events; however, iSpyMacCas9‐Combo‐*OsBBM1*‐gR2 produced higher and more robust editing outcomes even when compared with the iSpyMacCas9 construct (*P* < 0.05; Figure 3A). These results clearly suggest that strong activation of *OsBBM1* significantly improved genome editing efficiency, especially at the target sites that the standard iSpyMacCas9 system showed very low activity.

We further investigated the allele frequency at each target site to assess multiplexed genome editing outcomes by the top‐performing iSpyMacCas9‐Combo‐*OsBBM1*‐gR2 construct, in comparison to the standard iSpyMacCas9 system and an iSpyMacCas9‐Combo control without *OsBBM1*‐gR2. All these plants were generated from the standard rice transformation protocol with hormone‐supplemented regeneration medium. With iSpyMacCas9, most T0 lines showed biallelic editing at *OsGN1a* while editing at *OsGS3* and *OsROC5* was barely detected; only line no. 18 showed monoallelic editing at *OsGS3* (Figure 3B). In contrast, iSpyMacCas9‐Combo‐*OsBBM1*‐gR2 exhibited distinct editing profiles. At the *OsGN1a* target site, 80% of lines showed at least biallelic editing, like the results from iSpyMacCas9. Many potentially germline‐transmitted mutations were detected for *OsGS3* and *OsROC5*. For example, *OsROC5* was edited at chimeric (4/19), monoallelic (13/19), and biallelic (2/19) levels (Figure 3B; Table S2). Similarly, monoallelic editing of *OsGS3* was found in 7 out of 19 T0 lines (Figure 3B). In total, 15 out of the 19 T0 lines were double mutants and six were triple mutants (Figure 3B; Tables S2 and S3). In the HF regeneration condition, iSpyMacCas9‐Combo‐*OsBBM1*‐gR2 produced 10 double mutants (either monoallelic or biallelic) out of 21 T0 lines (Figure S4a). In contrast, few plants regenerated with the iSpyMacCas9 construct, and only editing at the *OsGN1a* target site was detected in such plants (Figure S4b). Together, these results indicate that *OsBBM1*‐gR2‐driven regeneration enriches the recovery of highly edited events under both hormone‐supplemented and HF regeneration conditions, although the detailed indel spectrum can vary between treatments.

**Figure 4:**
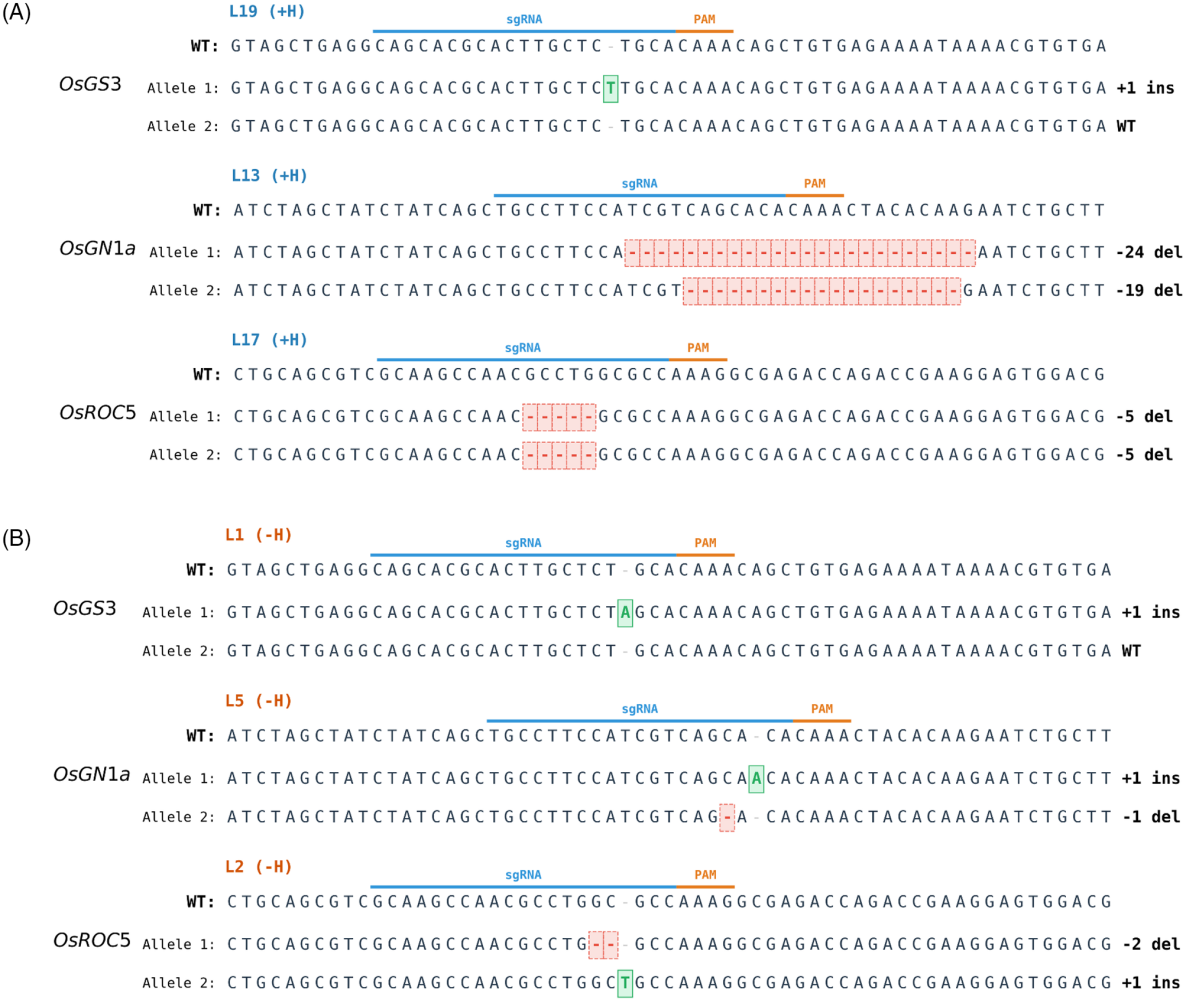
Representative genotypes of multiplexed edited lines recovered with iSpyMacCas9 CRISPR‐Combo under hormone and hormone‐free regeneration. (A) Representative edited T0 lines regenerated under hormone‐supplemented conditions. (B) Representative edited T0 lines regenerated under hormone‐free conditions. Allele schematics show the predominant edited alleles observed at each locus (top alleles), with deletions and insertions annotated as in the figure, illustrating multiplex genotypes recovered across regeneration conditions.

Across the targets, iSpyMacCas9‐Combo*‐OsBBM1*‐gR2 predominantly generated small indels at the expected cut sites, including single‐base insertions (e.g., in *OsGS3*) and deletions (e.g., in *OsGN1a* and *OsROC5*). Hormone‐supplemented lines included examples of larger and biallelic deletions at *OsGN1a* and *OsROC5*, similarly HF lines showed biallelic edited lines at both *OsGN1a* and *OsROC5* (Figure 4). Position‐resolved indel profiling across all three target sites confirmed that editing events remained concentrated at the expected cleavage position upstream of the PAM (Figure S5). Notably, at *OsGN1a*, the Combo system displayed a broader indel distribution and larger deletions spanning more positions upstream of the cut site compared with the no‐activation control. This pattern is consistent with increased recovery of edited events in lines with *OsBBM1* activation, further supporting the enhanced genome editing capacity of iSpyMacCas9‐Combo.

### iSpyMacCas9‐combo*‐OsBBM1*‐gR2 enables editing at challenging target sites

**Figure 5:**
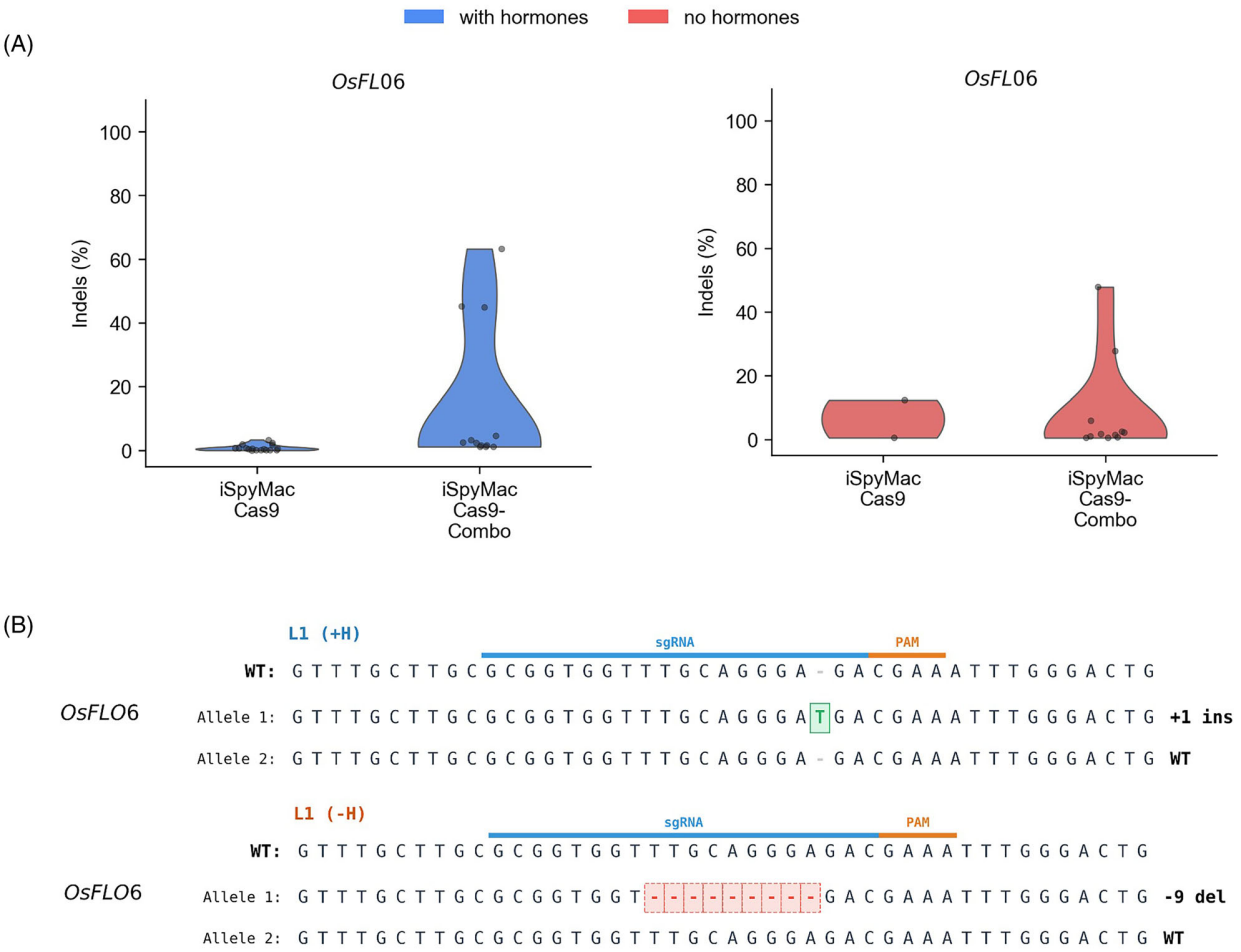
Improving iSpyMacCas9‐based genome editing at a challenging target site by simultaneous activation of *OsBBM1*. (A) Editing efficiencies (indel frequencies) at the *OsFLO6* target site in independent T0 lines regenerated under hormone‐supplemented or hormone‐free conditions, comparing iSpyMacCas9‐Combo to the non‐Combo control. Each dot represents one T0 line. (B) Representative genotypes at the *OsFLO6* locus from edited lines regenerated from both hormone and hormone‐free conditions.

To further test the reproducibility of *OsBBM1*‐gR2 in enriching iSpyMacCas9 genome editing efficiency in the Combo system, we targeted *OsFLO6* with one gRNA that showed very low editing efficiency based on our preliminary analysis. Again, both iSpyMacCas9 and iSpyMacCas9‐Combo constructs were made and compared under both hormone‐supplemented and HF conditions during rice regeneration. iSpyMacCas9 alone yielded only rare, low‐frequency (chimeric edits); whereas across both conditions, the iSpyMacCas9–Combo system produced a higher proportion of edited lines, indicating that the iSpyMacCas9‐Combo‐OsBBM1‐gR2 architecture improves recovery of edits at this challenging site (Figure 5A; Tables S4 and S5). Sequencing of representative alleles revealed indel signatures characteristic of iSpyMacCas9 activity, with edits concentrated around the predicted cleavage position (Figure 5B). Together, these results support the utility of iSpyMacCas9‐Combo for improving editing efficiency at difficult target loci.

## DISCUSSION

CRISPR‐Combo is an orthogonal molecular system that integrates CRISPRko and CRISPRa to enable simultaneous gene editing and transcriptional activation in plants (Pan et al., 2022; Pan & Qi, 2023). However, its reliance on the SpCas9 nuclease, which targets 5’‐NGG PAM sequences, restricts its utility in AT‐rich genomic regions such as promoter regions. To overcome this limitation, we investigated the use of Cas12b and iSpyMacCas9 nucleases, which recognize VTTV and NAAR PAMs, respectively, to broaden the applicability of CRISPR‐Combo in plants. In addition, we demonstrated the ability of these technologies to enable HF regeneration to enrich genome‐edited lines.

The CRISPR‐Cas12b‐Combo system did not show significant activation of *OsBBM1* in rice and hence did not result in any improvement in HF plant regeneration and genome editing. We argue that the highest *OsBBM1* activation achieved (~3‐fold) by the Cas12b‐Combo constructs was insufficient to trigger regeneration in these transgenic lines. As reported, the SpCas9‐Combo system could produce a 100‐fold upregulation of *OsBBM1* gene expression, and hence supported HF regeneration and enriched genome editing events (Pan et al., 2022). While it is possible to screen more gRNAs for higher *OsBBM1* activation, the low levels of transcriptional activation by the Cas12b system may be chiefly related to the relatively low efficiency of AaCas12b (Ming et al., 2020). These data suggest that the current CRISPR‐Cas12b‐Combo system is only suitable for mild transcriptional activation, which will constrain its potential use in plant genome engineering. Hence, this Cas12b‐Combo system warrants further improvement, such as the adoption of a highly efficient Cas12b nuclease.

This study used different gRNA expression architectures for Cas12b and iSpyMacCas9‐Combo systems. For Cas12b‐Combo, HH and HDV ribozymes (Gao & Zhao, 2014) were used to process a long RNA polymerase II‐derived polycistronic transcript to release Cas12b gRNAs, as in our previous work developing CRISPR‐Cas12b systems for plant genome editing (Ming et al., 2020). In contrast, the iSpyMacCas9‐Combo system used a tRNA‐based polycistronic gRNA expression system (Xie *et al*., 2015). tRNA‐based systems support the processing of multiple gRNAs from a single polycistronic transcript and may also enhance gRNA accumulation, as tRNA sequences contain intragenic RNA polymerase III promoter elements (He et al., 2026). Therefore, the higher activity of iSpyMacCas9‐Combo may have benefited from the use of tRNA‐based gRNA expression and processing architecture.

Considering the low efficiency of the Cas12b‐Combo system, we sought an alternative nuclease for A‐rich genomic regions. We previously developed an improved version of SpyMacCas9 (i.e., iSpyMacCas9) for genome editing in plants (Sretenovic et al., 2021). Increased gene editing efficiencies have already been suspected when CRISPR‐Cas systems are combined with the ectopic expression of transformation‐assisting morphogenic regulators, such as *WUSCHEL2* (*WUS2*) and *GROWTH REGULATORY FACTOR/GRF INTERACTING FACTOR* (*GRF‐GIF*), in recalcitrant plant species, such as sorghum and tropical maize (Hernandes‐Lopes *et al*., 2023; Che et al., 2022; Aesaert et al., 2022). Previously, we reported an association between *OsBBM1* activation as a morphogenic regulator and the improved targeted mutagenesis in rice (Pan et al., 2022). With iSpyMacCas9‐Combo, we further tested this correlation. We assessed the *OsBBM1* transcriptional activation efficiency by four different gRNAs via the newly developed CRISPR‐iSpyMacCas9‐Combo system, in which one gRNA – gR2 – showed around 12‐fold upregulation of *OsBBM1*. Notably, while the regeneration efficiency of gR2‐transformed calli was 38% for the control group (with hormones), we observed similar rates (42%) for these calli under HF conditions. In the previous CRISPR‐Combo paper (Pan et al., 2022), an average of 35.7% transformation efficiency was reported by SpCas9. Hence, it appears that iSpyMacCas9 can provide the same levels of HF regeneration as with SpCas9. Thus, it indicates that the iSpyMacCas9‐Combo system can also be leveraged for HF strategies in rice to enhance genome editing and expand the scope of CRISPR‐Combo systems to A‐rich genome contexts.

Indeed, our data support iSpyMacCas9‐Combo as a promising system for simultaneous genome editing and gene activation, whereas the latter requires targeting the A/T‐rich promoter regions. Furthermore, iSpyMacCas9‐Combo with *OsBBM1* activation was proven to be a better system than the standard iSpyMacCas9 system for genome editing in rice. This is a significant breakthrough because many orthogonal Cas9 nucleases, albeit with expanded targeting scope, often suffer from low editing efficiency. For example, our data showed that multiplexed monoallelic or biallelic genome editing at *OsGS3* and *OsROC5* was only made possible by using the iSpyMacCas9‐Combo‐*OsBBM1*‐gR2 strategy. Our success in rice will encourage future exploration of harnessing morphogenic gene activation to improve genome editing in other plants.

We envision continued expansion of iSpyMacCas9‐Combo systems in the future. Previously, we showed iSpyMacCas9 could be used for base editing (Sretenovic et al., 2021). Given CRISPR‐Combo‐CBE (cytosine base editor) was previously developed from the SpCas9D10A nickase and showed high C‐to‐T editing efficiency (Pan et al., 2022), iSpyMacCas9‐Combo‐CBE systems may be developed by testing different cytidine deaminases (Contiliani et al., 2025; Ren et al., 2021). Similarly, we envision the development of the CRISPR‐Combo‐PE (prime editing) system to allow for simultaneous precise genome editing and gene activation. Considering that prime editing is not very efficient in many plant species (Molla et al., 2021; Vats et al., 2024), it would be advantageous to develop CRISPR‐Combo‐PE systems with the potential to improve prime editing efficiency.

## CONCLUSIONS

Our study demonstrates that the CRISPR‐Combo toolkit can be expanded by incorporating alternative Cas nucleases, thereby broadening its applicability to AT‐rich genomic regions. While Cas12b‐Combo exhibited low efficiency, iSpyMacCas9‐Combo was highly efficient in simultaneous gene editing and activation. The iSpyMacCas9‐Combo‐OsBBM1‐gR2 system was established as a better genome editing system than iSpyMacCas9 alone. While our demonstration was limited to rice, this iSpyMacCas9 system is poised to be applied to other plant species to augment many potential genome engineering goals.

## MATERIALS AND METHODS

### Plant growth conditions

Rice (*Oryza sativa* sp. *Japonica* cv. Kitaake) plants were grown in the greenhouse at 29°C with a 16 h‐light/8 h‐dark cycle. Mature seeds were harvested and used for embryogenic callus induction for plant transformation.

### Assembly of CRISPR‐combo vectors

To generate the Cas12b‐Combo system, we amplified the MS2‐SunTag cassette alongside ZmUbi‐ScFv:sfGFP‐Terminator from pYPQ‐zCas9‐Act3.0 using Q5^®^ High‐Fidelity DNA Polymerase (NEB, USA) and cloned it into *Spe*I‐linearized pYPQ292 (Cas12b clone) using NEBuilder® HiFi DNA Assembly (NEB, USA). Similarly, we cloned the MS2‐SunTag and ZmUbi‐ScFv:sfGFP‐Terminator cassettes into *Sac*I‐linearized pYPQ166‐iSpyMacCas9, thereby generating the iSpyMacCas9‐Combo system. All the final vectors were checked by whole‐plasmid sequencing performed by Plasmidsaurus using Oxford Nanopore Technology with custom analysis and annotation.

To build multiplexed gRNA expression systems, gRNA oligos were annealed and phosphorylated using T4 Polynucleotide Kinase (New England Biolabs) under 37°C for 30 min, 95°C for 5 min and ramped down to 25°C (0.1°C/sec) and cloned into *BsmB*I‐digested pYPQ141‐ZmUbi‐RZ‐Aac.4 backbone with T4 DNA ligase. NEBuilder^®^ HiFi DNA Assembly (New England Biolabs) was used to bring *OsEPFL9*, *OsGS3*, and *OsBBM1* gRNAs in a single Gateway‐compatible gRNA expression vector. All vectors were constructed with SnapGene software and sequenced before transformation. All the final T‐DNA expression vectors were assembled by a Multisite Gateway cloning using an attL5‐attL2 gRNA entry clone, an attL1‐attR5 Cas entry clone, and an attR1‐attR2 destination vector pYPQ203 (Addgene no. 86207) using Gateway LR clonase II (Invitrogen).

### Rice stable transformation

All T‐DNA expression vectors were transformed into *Agrobacterium tumefaciens* (EHA105 strain). Rice embryogenic calli were transformed as previously described elsewhere (Pan & Qi, 2023), with minor modifications. After rice calli co‐cultivation, the calli were subdivided into control (with hormones) and HF. Control plates were supplemented with 2 mg L^−1^ 2,4‐D in coculture and selection media, 1 mg L^−1^ NAA, and 2 mg L^−1^ kinetin in regeneration I medium. To assess HF regeneration triggered by *OsBBM1* activation, no hormone was supplied into the HF plates with the above media.

### Mutation assessment by next‐generation sequencing

Genome editing outcomes in T0 plants were assessed by amplicon deep sequencing using a Hi‐TOM (High‐Throughput Tracking of Mutations) barcoding strategy (Liu et al., 2019). Leaf tissues were collected from hygromycin‐resistant rice seedlings and used for genomic DNA extraction using the CTAB method (Stewart & Via, 1993). Target genomic regions surrounding each CRISPR cut site were amplified by a two‐round PCR approach: the first round used gene‐specific primers with Hi‐TOM bridging sequences, and the second round added the sample‐specific barcodes and Illumina adapter sequences for multiplexed sequencing. Up to 48 barcoded amplicons per target were pooled, purified with the QIAQuick PCR purification Kit (QIAGEN), quantified by NanoDrop (Thermo Fisher Scientific), and sequenced on the Illumina MiSeq platform with 2 × 250 bp paired‐end reads (Amplifcon‐EZ service, Azenta Life Sciences).

Paired‐end FASTQ files were processed using a custom bioinformatics pipeline available as an interactive Google Colab notebook at: https://github.com/innocent250/ngs‐analysis‐pipeline. Briefly, reads were merged, demultiplexed by barcode, and editing outcome (indel frequencies, allele distributions) was quantified using CRISPResso2 in batch mode (Clement et al., 2019). Similar to our previous report (Zhang et al., 2021), T0 plant genotypes were classified based on indel frequency thresholds: wild‐type (WT, < 10%), chimeric (10–30%), monoallelic (30–70%), and biallelic (>70%).

### RNA extraction and gene expression analysis

Total RNA was extracted from calli and rice leaves by using the TRIzol protocol. To better represent each plate's callus population, 5–6 calli bulks were selected as individual samples for each construct. All the samples were treated with DNAse I (New England Biolabs), and approximately 500 ng of treated RNA was used for cDNA synthesis using SuperScript™ III Reverse Transcriptase (Thermo Fisher Scientific) following the manufacturers' suggestions. RT‐qPCR assay was performed using the AzuraQuant Green Fast qPCR Mix (Azura Genomics) in a CFX96 Touch Real‐Time PCR Detection System (Bio‐Rad). We used the rice *tubulin‐1* gene as a reference, and *OsBBM1* relative expression was determined by using the 2^–∆∆Ct^ method (Livak & Schmittgen, 2001).

### Statistical analysis

All data are presented as mean ± standard deviation (SD) unless otherwise stated. For RT‐qPCR analyses, *OsBBM1* relative expression was calculated using the 2^(−ΔΔCt) method with *OsTubulin‐1* as the reference gene. Differences in *OsBBM1* transcript levels between individual gRNA constructs and the no‐guide control were assessed by two‐tailed Student's *t*‐test using scipy.stats.ttest_ind, with *n* = 3 independent biological replicates per group (Figures 1C and 2E). For comparisons of indel frequencies across multiple constructs (Figure 3A), statistical significance was determined using a two‐sided‐Mann–Whitney U test (scipy.stats.mannwhitneyu), comparing each gRNA group to the iSpyMacCas9 control. Statistical significance thresholds were defined as: *P < 0.05, **P < 0.01, ***P < 0.001, and ****P < 0.0001. Figure 1 statistical analyses were performed in GraphPad Prism (version 10; GraphPad Software, San Diego, CA, USA). All other statistical analyses were performed in Python 3 using the SciPy library (Virtanen et al., 2020).

## Author Contributions

YQ conceptualized this research. YQ, IB, DFC, and FG designed the experiments. DFC generated the Cas12b‐Combo constructs, conducted rice transformation, and characterized the resulting lines for genome editing and target gene expression. IB generated the iSpyMacCas9‐CRISPR‐Combo constructs, conducted rice transformation, and did the molecular analysis of the resulting lines. CD supported the research in vector construction, rice transformation and genotyping. SC helped with the development of the manuscript. IB, DFC and YQ wrote the manuscript with input from other authors. All authors read and approved the final manuscript.

## Conflict of Interest

The authors declare no conflict of interest.

## Supporting information


**Figure S1.** Design principle of the Cas12b‐Combo system for simultaneous genome editing and transcriptional activation. Schematic of the Cas12b‐Combo system. Cas12b is guided by two sgRNA architectures: g1.0, a canonical 20‐nt sgRNA that directs Cas12b to induce a double‐strand break (DSB) for genome editing, and g2.0, a truncated 15‐nt sgRNA carrying a modified scaffold containing two MS2 aptamers. The MS2 aptamers recruit MCP fused to GCN4, which in turn recruits an scFV‐2 × TALE transcriptional activation domain (2TAD) complex, concentrating activators at the target site to drive transcriptional activation while Cas12b mediates editing.
**Figure S2.** iSpyMacCas9‐Combo architecture for simultaneous genome editing and transcriptional activation. (a) AlphaFold3‐based structural model illustrating the proposed basis for separating editing versus activation modes. A 20‐nt gRNA supports Cas9 recruitment and allows productive engagement with the target DNA that results in DSB (b), Schematic of the iSpyMacCas9‐Combo system that integrates targeted mutagenesis with programmable transcriptional activation. iSpyMacCas9 is co‐expressed with an MCP‐SunTag activator module, in which MCP is fused to a 10 × GCN4 SunTag to recruit an scFv complex carrying two TALE transcriptional activation domains (2TAD) (illustrated with sfGFP as a marker). Two guide designs are used: g1.0, a canonical 20‐nt sgRNA that directs iSpyMacCas9 to generate a double‐strand break (DSB) for genome editing, and g2.0, a truncated 15‐nt sgRNA containing a modified scaffold with two MS2 aptamers that recruit MCP. At the target locus, g1.0 mediates DSB formation (editing), while g2.0 nucleates MCP‐SunTag‐scFv‐2TAD recruitment to drive transcriptional activation of the targeted gene.
**Figure S3.** Workflow for generating and analyzing genome‐edited rice plants. Overview of the experimental pipeline. Candidate target genes are selected and corresponding gRNAs are designed, followed by plasmid construction. The resulting constructs are introduced into rice via *Agrobacterium*‐mediated transformation, and regenerated lines are recovered. Putative transgenic plants are then genotyped to assess editing outcomes, followed by downstream data analysis of mutation profiles and editing efficiency.
**Figure S4.** *OsBBM1* activation promotes recovery of heritable edits at multiplex target sites under hormone‐free regeneration. (a) Indel frequencies for individual T0 lines regenerated on hormone‐free medium following transformation with the iSpyMacCas9‐Combo construct carrying *OsBBM1‐gR2* activation. Each stacked bar represents one independent T0 line, partitioned by genotype class (WT, chimeric, monoallelic, biallelic), with dashed horizontal thresholds indicating the classification cutoffs. (b) Indel frequencies for T0 lines regenerated on hormone‐free medium using the same vector without *OsBBM1* activation, summarized as stacked bars for the indicated line sets and classified using the same genotype categories and thresholds as in (a).
**Figure S5.** Nucleotide‐resolution indel position profiles in T0 lines edited with iSpyMacCas9 or iSpyMacCas9‐Combo across hormone conditions. Each box spans the interquartile range (IQR; 25th–75th percentile) with the median line shown; whiskers extend to 1.5× IQR and individual dots represent single independent lines. The dashed vertical line marks the predicted cleavage site (3 bp upstream of the PAM); the red rectangle below the *x*‐axis delineates the 4‐bp iSpyMac PAM sequence; protospacer and PAM positions are labeled in blue and red, respectively. Only monoallelic and biallelic lines were included; raw CRISPResso indel frequencies are shown. Control lines are absent from some panels because no monoallelic or biallelic lines were recovered among controls for those target genes. (a) OsGN1a iSpyMacCas9, +Hormones (*n* = 19). (b) OsGN1a iSpyMacCas9‐Combo, +Hormones (*n* = 19). (c) OsGN1a iSpyMacCas9‐Combo, −Hormones (*n* = 21). (d) OsROC5 iSpyMacCas9‐Combo, +Hormones (*n* = 15). (e) OsROC5 iSpyMacCas9‐Combo, −Hormones (*n* = 8). (f) OsGS3 iSpyMacCas9‐Combo, +Hormones (*n* = 7). (g) OsGS3 iSpyMacCas9‐Combo, −Hormones (*n* = 2).
**Table S1.** iSpyMacCas9‐Combo transformation of rice calli.
**Table S2.** Zygosity classification of multiplexed T0 lines (with hormone).
**Table S3.** Zygosity classification of multiplexed T0 lines (hormone‐free).
**Table S4.** Zygosity classification of *OsFLO6* T0 lines (with hormone).
**Table S5.** Zygosity classification of OsFLO6 T0 lines (hormone‐free).
**Table S6.** Primers used in this study.
**Table S7.** gRNA oligos used in this study.

## Data Availability

The additional data supporting our findings are available in the Supplementary Materials of this article. The primers and gRNA oligos were summarized in Tables S6 and S7. The Google Colab pipeline used for Hi‐TOM amplicon sequencing analysis is publicly available at: https://github.com/innocent250/ngs‐analysis‐pipeline. The Gateway compatible vectors for A/T targeting Combo systems were deposited to Addgene: pYPQ‐AaCas12b‐Combo (#253552) and pYPQ‐iSpyMacCas9‐Combo (#253551).
