# Supplementary material for: Improving iSpyMacCas9 multiplex genome editing in rice by CRISPR‐combo‐mediated BBM1 activation
Source: Plant J. 2026 Jun 7;126(5):e70980. doi: 10.1111/tpj.70980 (PMC13242916; doi:10.1111/tpj.70980)
Supplement: Supplementary file 1 — Figure S1. Design principle of the Cas12b‐Combo system for simultaneous genome editing and transcriptional activation. Schematic of the Cas12b‐Combo system. Cas12b is guided by two sgRNA architectures: g1.0, a canonical 20‐nt sgRNA that directs Cas12b to induce a double‐strand break (DSB) for genome editing, and g2.0, a truncated 15‐nt sgRNA carrying a modified scaffold containing two MS2 aptamers. The MS2 aptamers recruit MCP fused to GCN4, which in turn recruits an scFV‐2 × TALE transcriptional activation domain (2TAD) complex, concentrating activators at the target site to drive transcriptional activation while Cas12b mediates editing. Figure S2. iSpyMacCas9‐Combo architecture for simultaneous genome editing and transcriptional activation. (a) AlphaFold3‐based structural model illustrating the proposed basis for separating editing versus activation modes. A 20‐nt gRNA supports Cas9 recruitment and allows productive engagement with the target DNA that results in DSB (b), Schematic of the iSpyMacCas9‐Combo system that integrates targeted mutagenesis with programmable transcriptional activation. iSpyMacCas9 is co‐expressed with an MCP‐SunTag activator module, in which MCP is fused to a 10 × GCN4 SunTag to recruit an scFv complex carrying two TALE transcriptional activation domains (2TAD) (illustrated with sfGFP as a marker). Two guide designs are used: g1.0, a canonical 20‐nt sgRNA that directs iSpyMacCas9 to generate a double‐strand break (DSB) for genome editing, and g2.0, a truncated 15‐nt sgRNA containing a modified scaffold with two MS2 aptamers that recruit MCP. At the target locus, g1.0 mediates DSB formation (editing), while g2.0 nucleates MCP‐SunTag‐scFv‐2TAD recruitment to drive transcriptional activation of the targeted gene. Figure S3. Workflow for generating and analyzing genome‐edited rice plants. Overview of the experimental pipeline. Candidate target genes are selected and corresponding gRNAs are designed, followed by plasmid construction. The result [file TPJ-126-0-s001.docx]

*Technical Advance*

**Improving iSpyMacCas9 multiplex genome editing in rice by CRISPR-Combo mediated *BBM1* activation**

Innocent Byiringiro^1*^, Danyel Fernandes Contiliani^1,2,3,*^, Colin Davies^1^, Filiz Gurel^1^, Silvana Creste^3^, Yiping Qi^1,4,#^

^1^ Department of Plant Science and Landscape Architecture, University of Maryland, College Park, MD, USA;

^2^ Graduate Program of Genetics, Ribeirao Preto Medical School, University of Sao Paulo, Ribeirao Preto, SP, Brazil;

^3^ Sugarcane Center, Agronomic Institute (IAC), Ribeirao Preto, SP, Brazil;

^4^ Institute of Bioscience and Biotechnology Research, University of Maryland, Rockville, MD, USA.

^*^ These authors contributed equally.

^#^ Corresponding author (yiping@umd.edu)

**Supplementary Figure 1-5**

**Supplementary Tables 1-7**


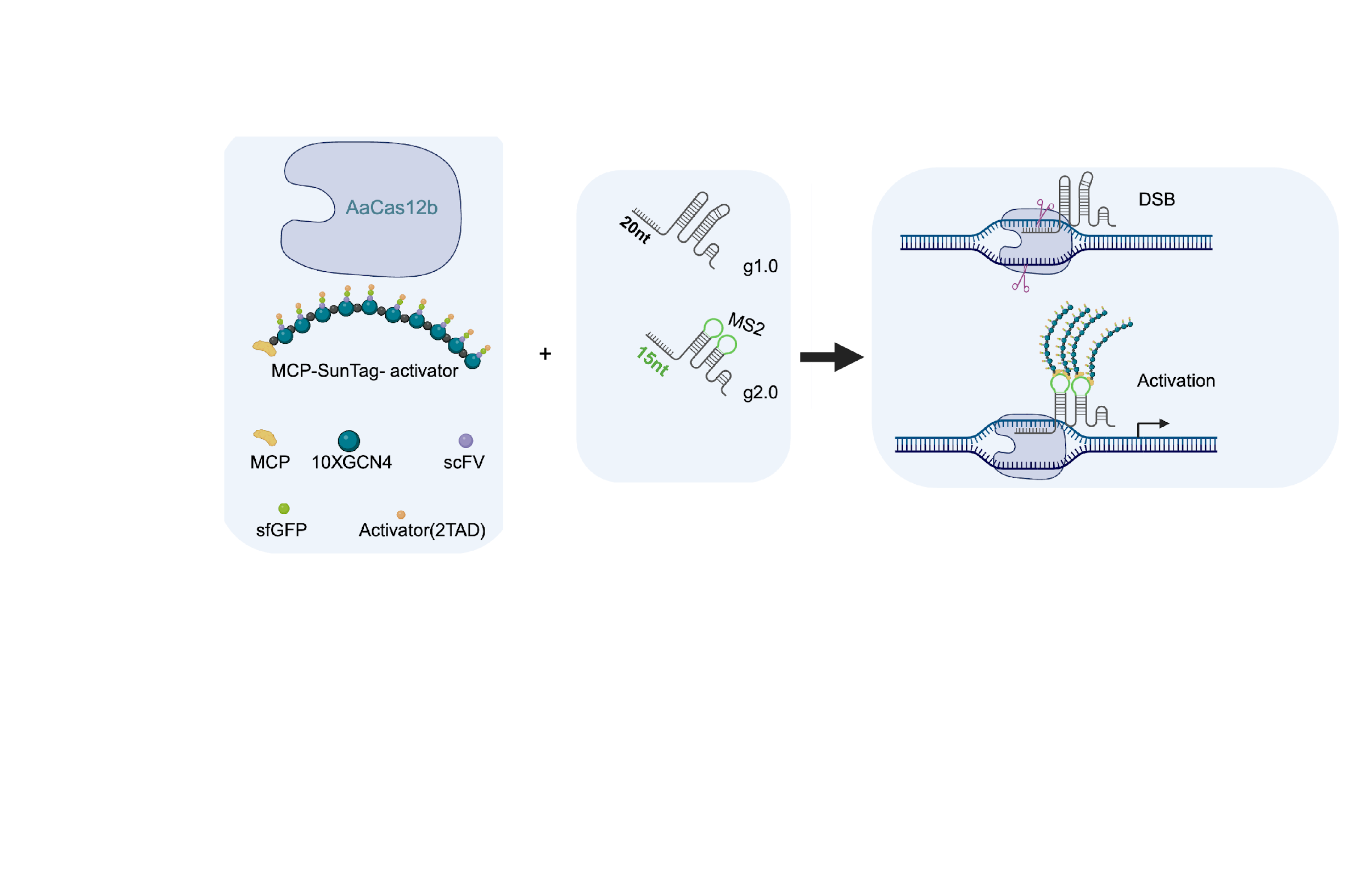


**Figure S1: Design principle of the Cas12b-Combo system for simultaneous genome editing and transcriptional activation.** Schematic of the Cas12b-Combo system. Cas12b is guided by two sgRNA architectures: g1.0, a canonical 20-nt sgRNA that directs Cas12b to induce a double-strand break (DSB) for genome editing, and g2.0, a truncated 15-nt sgRNA carrying a modified scaffold containing two MS2 aptamers. The MS2 aptamers recruit MCP fused to GCN4, which in turn recruits an scFV-2×TALE transcriptional activation domain (2TAD) complex, concentrating activators at the target site to drive transcriptional activation while Cas12b mediates editing.

**Figure S2: iSpyMacCas9-Combo architecture for simultaneous genome editing and transcriptional activation.** a, AlphaFold3-based structural model illustrating the proposed basis for separating editing versus activation modes. A 20-nt gRNA supports Cas9 recruitment and allows productive engagement with the target DNA that results in DSB b, Schematic of the iSpyMacCas9-Combo system that integrates targeted mutagenesis with programmable transcriptional activation. iSpyMacCas9 is co-expressed with an MCP-SunTag activator module, in which MCP is fused to a 10×GCN4 SunTag to recruit an scFv complex carrying two TALE transcriptional activation domains (2TAD) (illustrated with sfGFP as a marker). Two guide designs are used: g1.0, a canonical 20-nt sgRNA that directs iSpyMacCas9 to generate a double-strand break (DSB) for genome editing, and g2.0, a truncated 15-nt sgRNA containing a modified scaffold with two MS2 aptamers that recruit MCP. At the target locus, g1.0 mediates DSB formation (editing), while g2.0 nucleates MCP-SunTag-scFv-2TAD recruitment to drive transcriptional activation of the targeted gene.


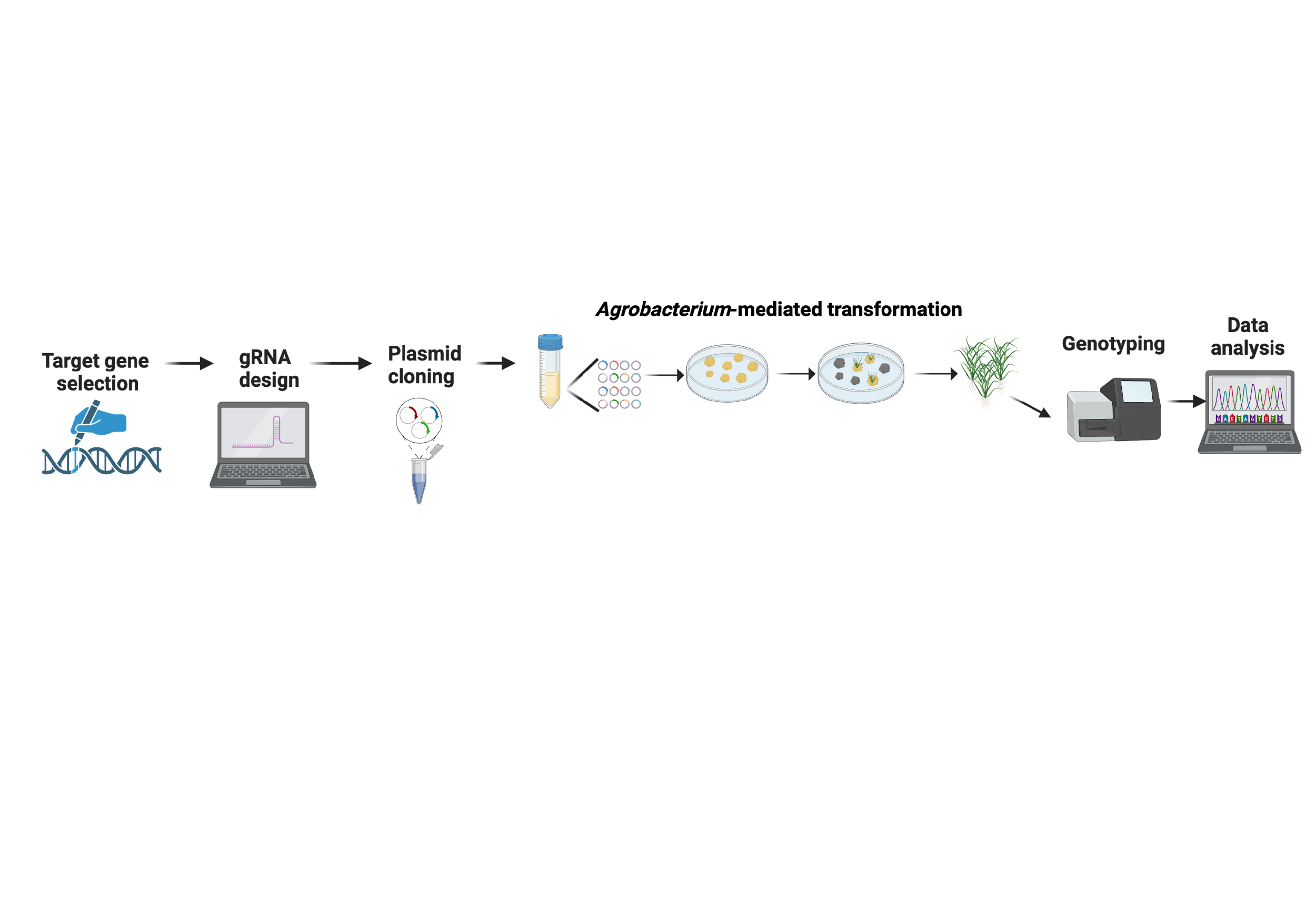


**Figure S3: Workflow for generating and analyzing genome-edited rice plants.** Overview of the experimental pipeline. Candidate target genes are selected and corresponding gRNAs are designed, followed by plasmid construction. The resulting constructs are introduced into rice via Agrobacterium-mediated transformation, and regenerated lines are recovered. Putative transgenic plants are then genotyped to assess editing outcomes, followed by downstream data analysis of mutation profiles and editing efficiency.

**Figure S4:  *OsBBM1* activation promotes recovery of heritable edits at multiplex target sites under hormone-free regeneration.** (a) Indel frequencies for individual T0 lines regenerated on hormone-free medium following transformation with the iSpyMacCas9-Combo construct carrying *OsBBM1-gR2* activation. Each stacked bar represents one independent T0 line, partitioned by genotype class (WT, chimeric, monoallelic, biallelic), with dashed horizontal thresholds indicating the classification cutoffs. (b) Indel frequencies for T0 lines regenerated on hormone-free medium using the same vector without *OsBBM1* activation, summarized as stacked bars for the indicated line sets and classified using the same genotype categories and thresholds as in (a).

a.

*OsGN1a* iSpyMacCas9 | +Hormones


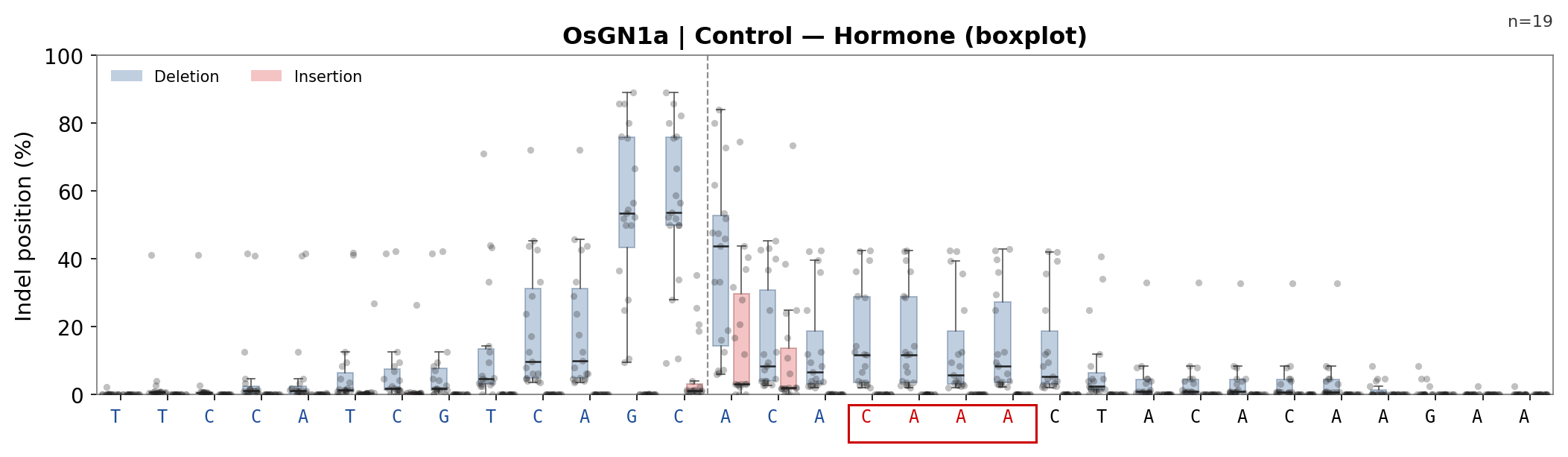


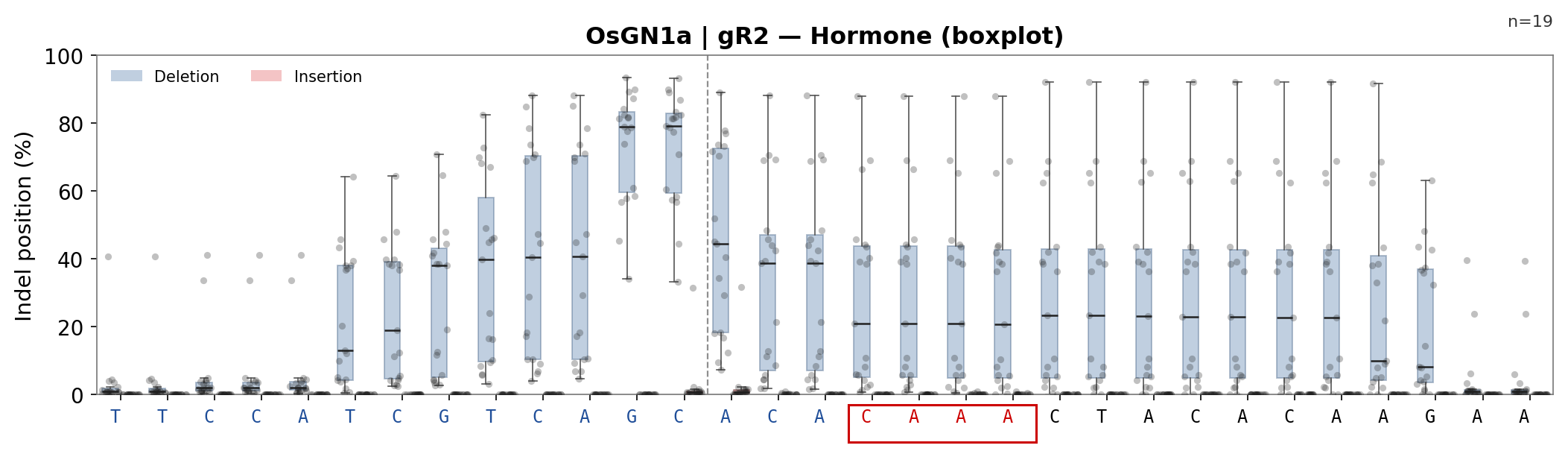


c.

b.

*OsGN1a* iSpyMacCas9-Combo | +Hormones

*OsGN1a* iSpyMacCas9-Combo | -Hormones


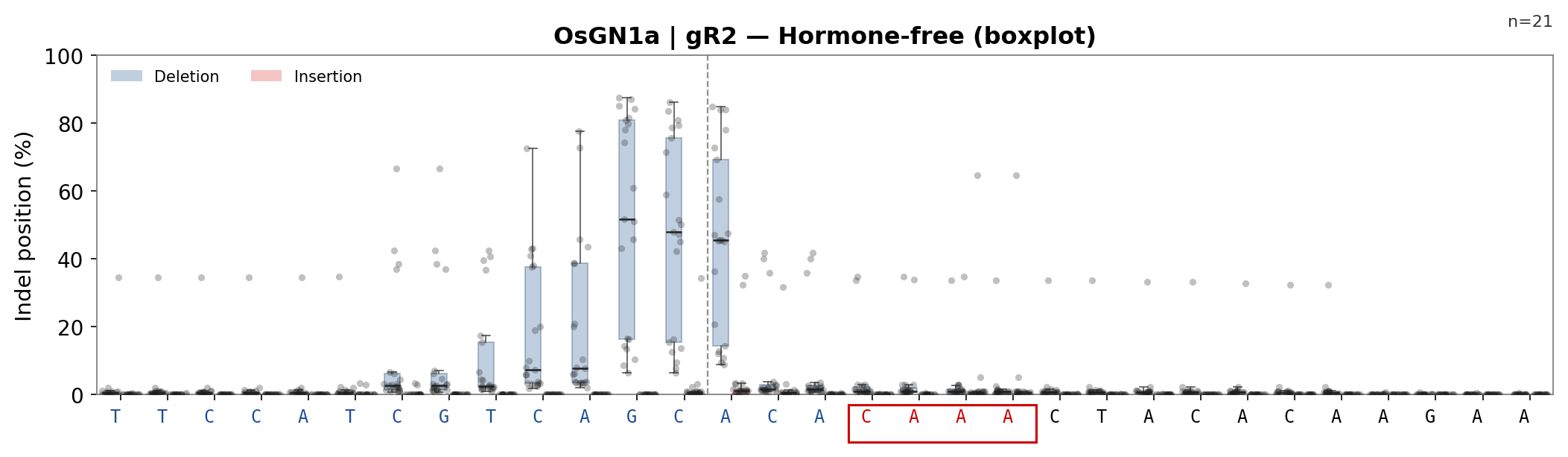


d.

*OsROC5* iSpyMacCas9-Combo | +Hormones


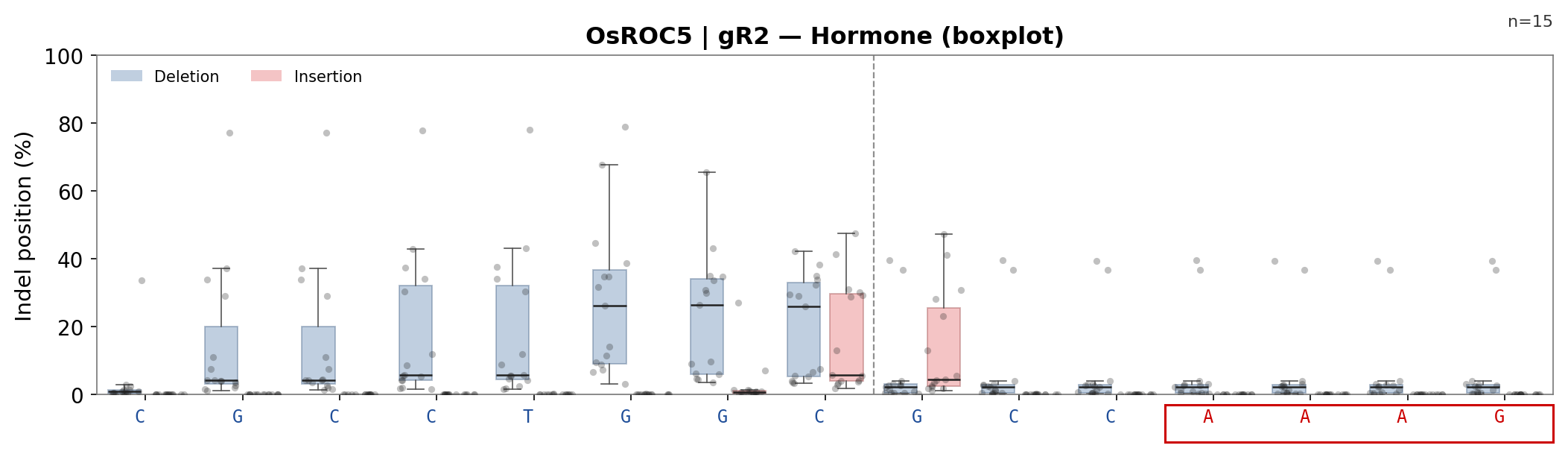


e.


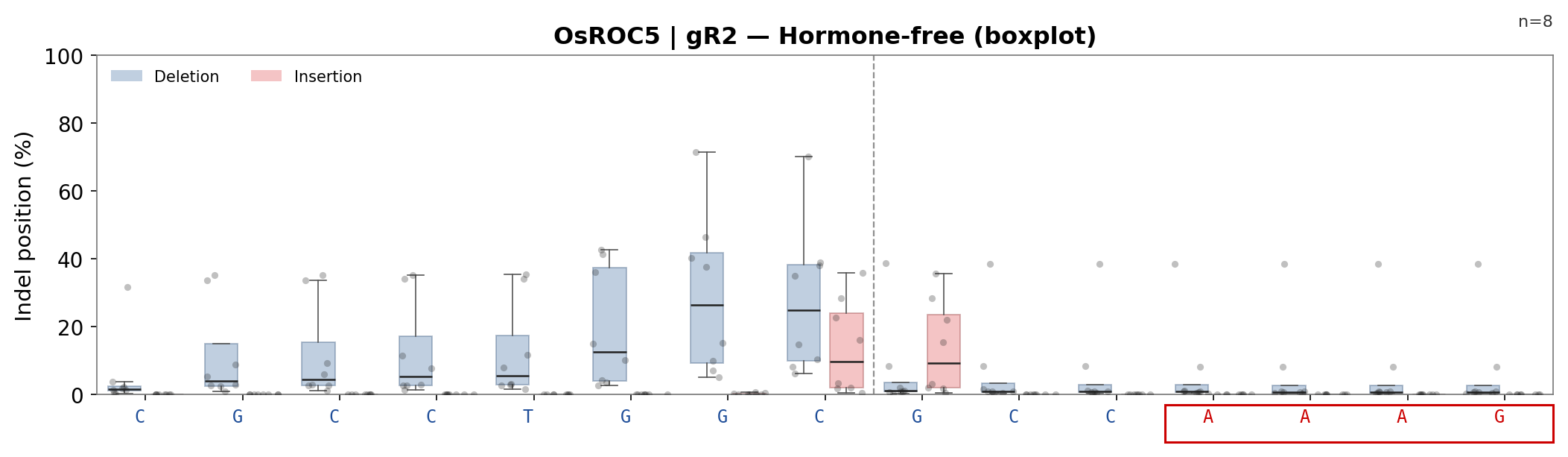


*OsROC5* iSpyMacCas9-Combo | -Hormones


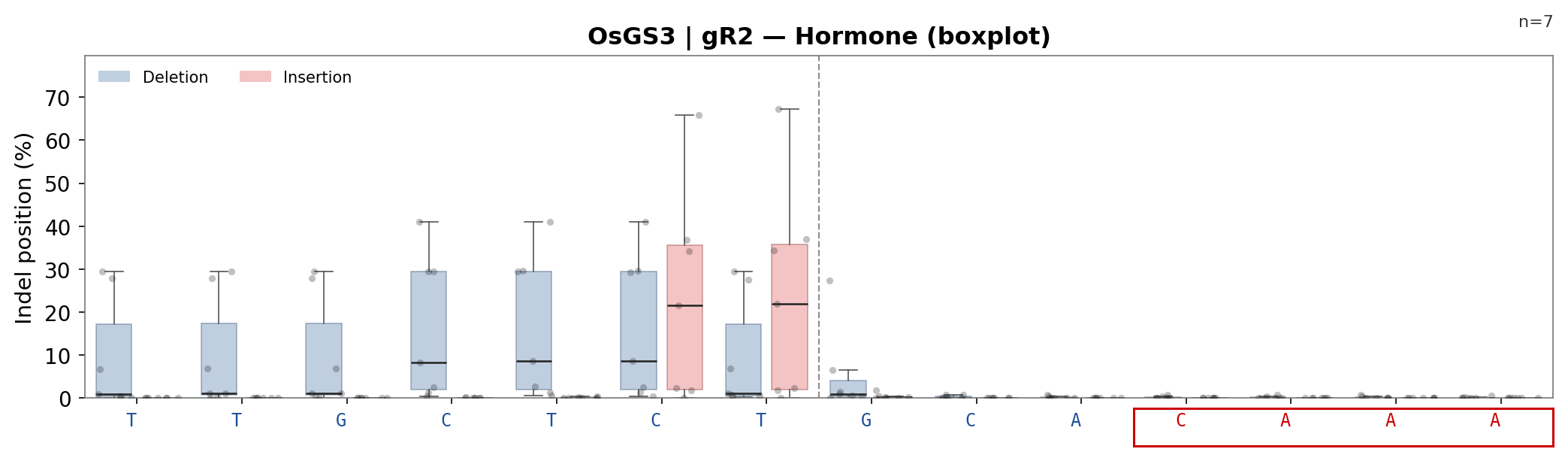


g.

f.

*OsGS3* iSpyMacCas9-Combo | +Hormones

*OsGS3* iSpyMacCas9-Combo | -Hormones


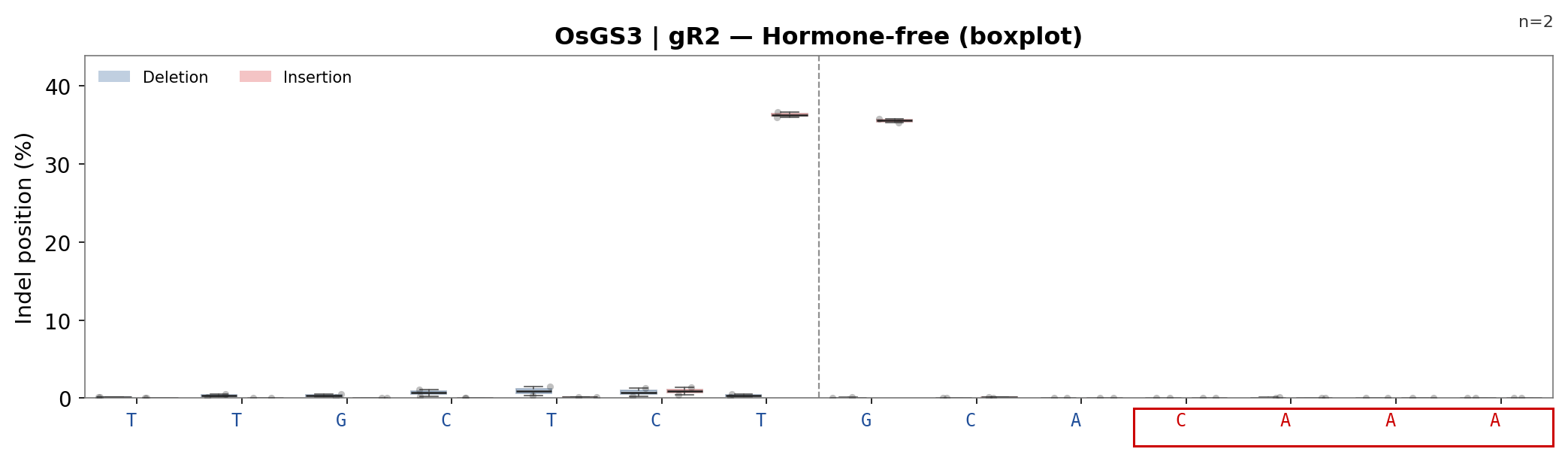


**Figure S5**: Nucleotide-resolution indel position profiles in T0 lines edited with iSpyMacCas9 or iSpyMacCas9-Combo across hormone conditions. Each box spans the interquartile range (IQR; 25th-75th percentile) with the median line shown; whiskers extend to 1.5× IQR and individual dots represent single independent lines. The dashed vertical line marks the predicted cleavage site (3 bp upstream of the PAM); the red rectangle below the x-axis delineates the 4-bp iSpyMac PAM sequence; protospacer and PAM positions are labelled in blue and red, respectively. Only monoallelic and biallelic lines were included; raw CRISPResso indel frequencies are shown. Control lines are absent from some panels because no monoallelic or biallelic lines were recovered among controls for those target genes. (a) OsGN1a iSpyMacCas9, +Hormones (n = 19). (b) OsGN1a iSpyMacCas9-Combo, +Hormones (n = 19). (c) OsGN1a iSpyMacCas9-Combo, −Hormones (n = 21). (d) OsROC5 iSpyMacCas9-Combo, +Hormones (n = 15). (e) OsROC5 iSpyMacCas9-Combo, −Hormones (n = 8). (f) OsGS3 iSpyMacCas9-Combo, +Hormones (n = 7). (g) OsGS3 iSpyMacCas9-Combo, −Hormones (n = 2).

**Table S1. iSpyMacCas9-Combo transformation of rice calli.**

| T-DNA | Activation gRNA | Treatment | Calli transformed | Regenerated plants | Efficiency |
| --- | --- | --- | --- | --- | --- |
| pLR5382 | gR1 | HF | 50 | 7 | 14% |
|  |  | H | 50 | 25 | 50% |
| pLR5383 | gR2 | HF | 50 | 21 | 42% |
|  |  | H | 50 | 19 | 38% |
| pLR5384 | gR3 | HF | 50 | 5 | 10% |
|  |  | H | 50 | 19 | 38% |
| pLR5385 | gR4 | HF | 50 | 12 | 24% |
|  |  | H | 50 | 12 | 24% |
| pLR5390 | - | HF | 50 | 2 | 4% |
|  |  | H | 50 | 19 | 38% |

*HF - Hormone-free treatment; H - Hormone treatment. Each row represents a separate T-DNA construct transformed into ~50 rice calli. “Activation gRNA” indicates which OsBBM1 guide (if any) was included in the construct. HF, hormone-free regeneration; H, hormone-supplemented regeneration. The number of regenerated plants and the percentage regeneration efficiency are shown for each treatment. The gR2 construct achieved the highest hormone-free regeneration (42%, matching its performance with hormones). The no-activation control (pLR5390) had minimal regeneration without hormones (4%). (HF: hormone-free treatment; H: hormone treatment.*

**Table S2. Zygosity classification of multiplexed T0 lines (with hormone).**

| **OsBBM1 activation gRNA** | **Total Lines** | ***OsGN1a*** | | | | ***OsGS3*** | | | | ***OsROC5*** | | | |
| --- | --- | --- | --- | --- | --- | --- | --- | --- | --- | --- | --- | --- | --- |
|  |  | WT n (%) | Chimeric n (%) | Monoallelic n (%) | Biallelic n (%) | WT n (%) | Chimeric n (%) | Monoallelic n (%) | Biallelic n (%) | WT n (%) | Chimeric n (%) | Monoallelic n (%) | Biallelic n (%) |
| - | 19 | 0 (0%) | 1 (5%) | 1 (5%) | 17 (89%) | 18 (95%) | 0 (0%) | 1 (5%) | 0 (0%) | 19 (100%) | 0 (0%) | 0 (0%) | 0 (0%) |
| gR1 | 24 | 2 (8%) | 10 (42%) | 7 (29%) | 5 (21%) | 24 (100%) | 0 (0%) | 0 (0%) | 0 (0%) | 20 (83%) | 4 (17%) | 0 (0%) | 0 (0%) |
| gR2 | 19 | 0 (0%) | 0 (0%) | 3 (16%) | 16 (84%) | 9 (47%) | 3 (16%) | 7 (37%) | 0 (0%) | 0 (0%) | 4 (21%) | 13 (68%) | 2 (11%) |
| gR3 | 19 | 0 (0%) | 13 (68%) | 4 (21%) | 2 (11%) | 18 (95%) | 1 (5%) | 0 (0%) | 0 (0%) | 19 (100%) | 0 (0%) | 0 (0%) | 0 (0%) |
| gR4 | 12 | 0 (0%) | 8 (67%) | 4 (33%) | 0 (0%) | 12 (100%) | 0 (0%) | 0 (0%) | 0 (0%) | 12 (100%) | 0 (0%) | 0 (0%) | 0 (0%) |

*Zygosity thresholds: WT < 10%; Chimeric: 10%-30%; Monoallelic: 30%-70%; Biallelic: > 70%*

**Table S3. Zygosity classification of multiplexed T0 lines (hormone-free).**

| **OsBBM1 activation gRNA** | **Total Lines** | ***OsGN1a*** | | | | ***OsGS3*** | | | | ***OsROC5*** | | | |
| --- | --- | --- | --- | --- | --- | --- | --- | --- | --- | --- | --- | --- | --- |
|  |  | WT n (%) | Chimeric n (%) | Monoallelic n (%) | Biallelic n (%) | WT n (%) | Chimeric n (%) | Monoallelic n (%) | Biallelic n (%) | WT n (%) | Chimeric n (%) | Monoallelic n (%) | Biallelic n (%) |
| - | 2 | 0 (0%) | 0 (0%) | 1 (50%) | 1 (50%) | 2 (100%) | 0 (0%) | 0 (0%) | 0 (0%) | 2 (100%) | 0 (0%) | 0 (0%) | 0 (0%) |
| gR1 | 7 | 1 (14%) | 4 (57%) | 0 (0%) | 2 (29%) | 7 (100%) | 0 (0%) | 0 (0%) | 0 (0%) | 5 (71%) | 2 (29%) | 0 (0%) | 0 (0%) |
| gR2 | 21 | 0 (0%) | 0 (0%) | 2 (10%) | 19 (90%) | 18 (86%) | 1 (5%) | 2 (10%) | 0 (0%) | 4 (19%) | 9 (43%) | 5 (24%) | 3 (14%) |
| gR3 | 5 | 0 (0%) | 3 (60%) | 0 (0%) | 2 (40%) | 5 (100%) | 0 (0%) | 0 (0%) | 0 (0%) | 4 (80%) | 1 (20%) | 0 (0%) | 0 (0%) |
| gR4 | 12 | 0 (0%) | 2 (17%) | 3 (25%) | 7 (58%) | 12 (100%) | 0 (0%) | 0 (0%) | 0 (0%) | 12 (100%) | 0 (0%) | 0 (0%) | 0 (0%) |

*Zygosity thresholds: WT < 10%; Chimeric: 10%-30%; Monoallelic: 30%-70%; Biallelic: > 70%*

**Table S4. Zygosity classification of *OsFLO6* T0 lines (with hormone).**

| **Total Lines** | **WT n (%)** | **Chimeric n (%)** | **Monoallelic n (%)** | **Biallelic n (%)** |
| --- | --- | --- | --- | --- |
| 12 | 9 (75%) | 0 (0%) | 3 (25%) | 0 (0%) |

*Zygosity thresholds: WT < 10%; Chimeric: 10%-30%; Monoallelic: 30%-70%; Biallelic: > 70*

**Table S5. Zygosity classification of OsFLO6 T0 lines (hormone-free).**

| **Total Lines** | **WT n (%)** | **Chimeric n (%)** | **Monoallelic n (%)** | **Biallelic n (%)** |
| --- | --- | --- | --- | --- |
| 11 | 9 (82%) | 1 (9%) | 1 (9%) | 0 (0%) |

*Zygosity thresholds: WT < 10%; Chimeric: 10%-30%; Monoallelic: 30%-70%; Biallelic: > 70%*

**Table S6. Primers used in this study**

| **Primer Name** | **Sequence (5’-3’)** |
| --- | --- |
| Hi-Tom-*OsEPFL9*-F | ggagtgagtacggtgtgcATGCTTGCCCCACATCTACC |
| Hi-Tom-*OsEPFL9*-R | gagttggatgctggatggTCTTCAGGTAATGTTTGGTGAG |
| Hi-Tom-*OsGS3*-F (Cas12b) | ggagtgagtacggtgtgcCCTGGTGAACTTCGTCGATTGT |
| Hi-Tom-*OsGS3*-R (Cas12b) | gagttggatgctggatggTCGCTTCTCCGATGAACTGC |
| Hi-Tom-*OsGN1a*-F | ggagtgagtacggtgtgcCAGTCCTTGTCACACAGATC |
| Hi-Tom-*OsGN1a*-R | gagttggatgctggatggCCGAGGTCGCCGAGGTCGTC |
| Hi-Tom-*OsGS3*-F (iSpyMac) | ggagtgagtacggtgtgcCCCACAAAACCATCAACTTG |
| Hi-Tom-*OsGS3*-R (iSpyMac) | gagttggatgctggatggCGACACGGACTCTTCGTTAA |
| Hi-Tom-*OsROC5*-F | ggagtgagtacggtgtgcAGAGCTCTCATCTGATGTAT |
| Hi-Tom-*OsROC5*-R | gagttggatgctggatggTTGAGCTCGTTTTAGATGGA |
| Hi-Tom-*OsFLO6*-F | ggagtgagtacggtgtgcTGCTCCCTTGGGGTGTTCGT |
| Hi-Tom-*OsFLO6*-R | gagttggatgctggatggCCTCTGCAGCCTGGTAAGCA |
| qRT-PCR_*OsBBM1*-F | CAGCGGACGTCCATCTACCG |
| qRT-PCR_*OsBBM1*-R | GGGCTTCATACCTTCCTGTCCA |

**Table S7. gRNA oligos used in this study**

| **System** | **Activity** | **Oligo Name** | **Sequence (5’-3’)** |
| --- | --- | --- | --- |
| **Cas12b-Combo** | **Editing (g1.0)** | *OsEPFL9*-sgRNA02-F | ggcacCAATCAAGGGCACCATGGCA |
|  |  | *OsEPFL9*-sgRNA02-R | ggccTGCCATGGTGCCCTTGATTGg |
|  |  | *OsGS3*-sgRNA02-F | ggcacATCGGAAGAACTCCTGATCC |
|  |  | *OsGS3*-sgRNA02-R | ggccGGATCAGGAGTTCTTCCGATg |
|  | **Activation (g2.0)** | *OsBBM1*-gR1-AaCas12b-F | ggcacCTGAGACTAGAAAGG |
|  |  | *OsBBM1*-gR1-AaCas12b-R | ggccCCTTTCTAGTCTCAGg |
|  |  | *OsBBM1*-gR2-AaCas12b-F | ggcacGGGTTATCAACACAG |
|  |  | *OsBBM1*-gR2-AaCas12b-R | ggccCTGTGTTGATAACCCg |
|  |  | *OsBBM1*-gR3-AaCas12b-F | ggcacAAGTGCATGCCATGC |
|  |  | *OsBBM1*-gR3-AaCas12b-R | ggccGCATGGCATGCACTTg |
|  |  | *OsBBM1*-gR4-AaCas12b-F | ggcacGTATCAAAACATCCG |
|  |  | *OsBBM1*-gR4-AaCas12b-R | ggccCGGATGTTTTGATACg |
|  |  | *OsBBM1*-gR5-AaCas12b-F | ggcacGCCGGTGAAGCAATT |
|  |  | *OsBBM1*-gR5-AaCas12b-R | ggccAATTGCTTCACCGGCg |
| **iSpyMacCas9-Combo** | **Editing (g1.0)** | *OsGS3*-sg3-F | tgcaCAGCACGCACTTGCTCTGCA |
|  |  | *OsGS3*-sg3-R | aaacTGCAGAGCAAGTGCGTGCTG |
|  |  | *OsGN1a*-sg3-F | tgcaTGCCTTCCATCGTCAGCACA |
|  |  | *OsGN1a*-sg3-R | aaacTGTGCTGACGATGGAAGGCA |
|  |  | *OsROC5*-sg3-F | tgcaGCAAGCCAACGCCTGGCGCC |
|  |  | *OsROC5*-sg3-R | aaacGGCGCCAGGCGTTGGCTTGC |
|  |  | *OsFLO6-sg-F* | tgcaGCGGTGGTTTGCAGGGAGAC |
|  |  | *OsFLO6-sg-R* | aaacGTCTCCCTGCAAACCACCGC |
|  | **Activation (g2.0)** | *OsBBM1*-gR1-F | tgcaGCGTGTGTGTGCATG |
|  |  | *OsBBM1*-gR1-R | aaacCATGCACACACACGC |
|  |  | *OsBBM1*-gR2-F | tgcaGCAGAGGTAGAGAGA |
|  |  | *OsBBM1*-gR2-R | aaacTCTCTCTACCTCTGC |
|  |  | *OsBBM1*-gR3-F | tgcaAGGGCTTGCTGCCCA |
|  |  | *OsBBM1*-gR3-R | aaacTGGGCAGCAAGCCCT |
|  |  | *OsBBM1*-gR4-F | tgcaATTTGCTGAGACTAG |
|  |  | *OsBBM1*-gR4-R | aaacCTAGTCTCAGCAAAT |
